# Life events and escape in conversion disorder

**DOI:** 10.1017/S0033291716000714

**Published:** 2016-07-05

**Authors:** T. R. Nicholson, S. Aybek, T. Craig, T. Harris, W. Wojcik, A. S. David, R. A Kanaan

**Affiliations:** 1 Section of Cognitive Neuropsychiatry, Institute of Psychiatry Psychology & Neuroscience, King's College, London, UK; 2 Laboratory for Behavioral Neurology and Imaging of Cognition, Fundamental Neurosciences Department, Geneva University, Geneva, Switzerland; 3 Health Services Research Department, Institute of Psychiatry Psychology & Neuroscience, King's College, London, UK; 4 Department of Psychological Medicine, Institute of Psychiatry Psychology & Neuroscience, King's College, London, UK; 5 Department of Psychiatry, University of Melbourne, Austin Health, Heidelberg, Victoria, Australia

**Keywords:** Conversion disorder, functional neurological disorder, life events, stress, trauma

## Abstract

**Background:**

Psychological models of conversion disorder (CD) traditionally assume that psychosocial stressors are identifiable around symptom onset. In the face of limited supportive evidence such models are being challenged.

**Method:**

Forty-three motor CD patients, 28 depression patients and 28 healthy controls were assessed using the Life Events and Difficulties Schedule in the year before symptom onset. A novel ‘escape’ rating for events was developed to test the Freudian theory that physical symptoms of CD could provide escape from stressors, a form of ‘secondary gain’.

**Results:**

CD patients had significantly more severe life events and ‘escape’ events than controls. In the month before symptom onset at least one severe event was identified in 56% of CD patients – significantly more than 21% of depression patients [odds ratio (OR) 4.63, 95% confidence interval (CI) 1.56–13.70] and healthy controls (OR 5.81, 95% CI 1.86–18.2). In the same time period 53% of CD patients had at least one ‘high escape’ event – again significantly higher than 14% in depression patients (OR 6.90, 95% CI 2.05–23.6) and 0% in healthy controls. Previous sexual abuse was more commonly reported in CD than controls, and in one third of female patients was contextually relevant to life events at symptom onset. The majority (88%) of life events of potential aetiological relevance were not identified by routine clinical assessments. Nine per cent of CD patients had no identifiable severe life events.

**Conclusions:**

Evidence was found supporting the psychological model of CD, the Freudian notion of escape and the potential aetiological relevance of childhood traumas in some patients. Uncovering stressors of potential aetiological relevance requires thorough psychosocial evaluation.

## Introduction

Conversion disorder (CD), previously known as hysteria, describes neurological symptoms, such as weakness or sensory loss, that are not thought to be due to neurological disease. Aetiological explanations for CD have, historically, been dominated by psychological models, particularly those of Sigmund Freud. Put simply, Freud proposed that the ‘unbearable affect’ of stressors was ‘converted’ into physical symptoms, a process which itself reduced such affect, and he called this ‘primary gain’ (Breuer & Freud, 1895). Further, he proposed that the ensuing physical manifestations would also reduce the stressor by changing the individual's circumstances (e.g. weakness resulting in being unable to return to work where there is bullying) which he termed ‘secondary gain’ and ICD-10 supports this notion stating that ‘Assessment of the patient's mental state and social situation usually suggests that the disability resulting from the loss of functions is helping the patient to escape from an unpleasant conflict’ (WHO, 1992). Other key features of Freud's theories were the mechanism of repression (of awareness of severity, or even the presence, of stressors) and the relevance of severe early traumas, particularly sexual abuse. In the face of lack of supportive evidence the influence of these theories has been steadily waning with less and less explicit mention of such theories with successive iterations of diagnostic criteria. Furthermore, the once essential diagnostic criterion for the identification of stressors around the time of symptom onset has been removed in DSM-5 (APA, 2013) and the psychological model more broadly has been challenged, primarily on the basis of a current lack of evidence for an association between stressors and the disorder (Stone & Edwards, 2011).

However, there have been no controlled studies of the rates of life events in common forms of CD (e.g. weakness or paralysis) using comprehensive clinician-rated (rather than self-report) assessments (Roelofs & Spinhoven, 2007; Nicholson *et al.* 2011) such as the Life Events and Difficulties Schedule (LEDS; Brown & Harris, 1978, 1989). The LEDS has, however, been used to study less common variants of CD such as globus pharyngis, the feeling of a persistent lump in the throat, patients with which experienced increased rates of severe life events compared to general otolaryngology controls (Harris *et al.* 1996). The LEDS also revealed elevated rates of severe events in functional dysphonia for the month before symptom onset. Intriguingly over half of the dysphonia patients had an event involving ‘conflict over speaking out’ providing evidence for secondary gain (House & Andrews, 1988) which has since been replicated in a larger sample including organic as well as healthy controls (Baker *et al.* 2013). There is also evidence from the related condition of somatization disorder for the relevance of secondary gain in at least symptom maintenance (Craig *et al.* 1994). There is also robust evidence for elevated rates of historical stressors such as childhood abuse, particularly for sexual abuse in the seizure variant of CD, with a meta-analysis of 16 contrasts giving a pooled odds ratio (OR) of 2.94 [95% confidence interval (CI) 2.29–3.77] compared to various control groups (Sharpe & Faye, 2006). A prospective study of 50 patients with possible CD, using non-standardized assessments, provided some preliminary evidence for the predictive value of both stressors prior to symptom onset and secondary gain which they defined as ‘gain from symptom solves conflict of precipitating stress’ (Raskin *et al.* 1966)

Finally, we have recently found evidence for the relevance of stressful life events in motor CD in a fMRI study where the neural correlates of recall of stressors of potential aetiological relevance, when compared to events of matched severity, revealed differential activation in areas involved in emotion and memory control with associated changes in motor areas, providing a possible ‘conversion’ mechanism (Aybek *et al.* 2014).

This study tested the psychological model of CD by thoroughly assessing life events (using the LEDS) in motor CD in comparison to both healthy and psychiatric controls. We also examined the specificity of certain events and their relatedness to symptoms, namely those providing ‘escape’ from stressors, a form of secondary gain.

## Method

### Subjects

Forty-three patients with motor CD with symptom onset within the last 2 years (to maximize stressor recall) were recruited consecutively from secondary (neurology and consultation-liaison psychiatry) and tertiary (neuropsychiatry) care services. Case definition was based on DSM-IV clinical diagnosis but did not require identification of psychological stressors around the time of onset of the disorder. Diagnosis was confirmed by an experienced neurologist and/or neuropsychiatrist on the basis of chart review. Twenty-eight healthy controls were recruited contemporaneously from primary-care registers in the same geographical area (South-East London) using stratified random-sampling, matching for age and gender. Twenty-eight major depression patients recruited from secondary-care settings for a previous study in the same location provided additional comparison data using the same measures (Brown *et al.* 1994).

Participants were excluded if non-fluent English speakers or if they had a current or historical neurological disorder, somatoform disorder or major mental disorder (e.g. psychotic disorder). Written informed consent was gained at inclusion and the study was approved by the UK National Research Ethics Service (reference 07/H0805/33).

### Ethical standards

The authors assert that all procedures contributing to this work comply with the ethical standards of the relevant national and institutional committees on human experimentation and with the Helsinki Declaration of 1975, as revised in 2008.

### Life events assessment

The LEDS (Brown & Harris, 1978) was used to assess stressors in the form of ‘events’ (discrete episodes) and ‘difficulties’ (lasting at least 2 weeks). The study period for CD and depression patients was the year before symptom onset. For healthy controls the 2 years prior to interview date were studied to minimize differences in time from interview to life events, and therefore reliability of recall, compared to patients. All interviewers underwent formal training in LEDS assessment and consensus panels, blinded to group status, rated events for each subject. The LEDS enquires about different life domains, such as health, accommodation and employment in a semi-structured format which takes 2–4 h to deliver. As such it is a particularly comprehensive instrument, has high inter-rater reliability, high levels of concordance between subject and independent informant accounts (Brown & Harris, 1978; Tennant *et al.* 1979) and can accurately detect stressors 5 years after their occurrence (Neilson *et al.* 1989). We aimed to assess the nature (particularly severity) and timing of stressors in the study periods.

‘Severe’ events were classified, according to standard LEDS methodology, as those with long-term threat (severity) scores of either 1 (marked threat/severity) or 2 (moderate threat/severity, focused on the respondent) on a scale out of 4; scores of 3 (some threat) or 4 (little or no threat) were classified as non-severe as were moderate events focused on persons other than the respondent. Event severity is scored both ‘objectively’ by the consensus panel and ‘subjectively’ according to the interviewer's impression of the subject's description. Instances where subjective levels of severity were judged lower than objective ratings reported were termed ‘under-reported’ and used as a proxy for ‘repression’. Difficulties were rated similarly for severity according to standard methodology; 4 = low moderate, and 1, 2 or 3 = severe.

A novel rating for ‘escape’ (potential) was developed (Aybek *et al.* 2014) which estimates the extent to which the impact of a stressor might be ameliorated by being ill with neurological symptoms such as weakness, e.g. providing escape from a bullying boss/abusive parent or preventing a partner from leaving, consistent with the aforementioned preliminary study (Raskin *et al.* 1966). Escape ratings were as follows; 0 = no, 1 = some, 2 = moderate; 3 = marked. Ratings of 1, 2 or 3 were therefore ‘any’ and 2 or 3 were ‘high’ escape. With the example of the partner leaving, or threatening to leave, other contextual information can influence the escape rating such as the partner being uncaring and therefore less likely to be influenced by the subject becoming ill. Note that the loss of a partner in another way, e.g. dying of a heart attack, despite being very stressful, would have no significant escape potential, as becoming ill would not alleviate the stressor.

For CD patients the potential aetiological relevance of events was assessed by severity, closeness to the onset of the disorder and escape rating and those scoring highly on all three, we label these ‘key’ events. These factors guided the consensus panel in a final psychological formulation. The LEDS includes questions on past as well as recent history of, sexual and physical abuse and if this was disclosed, further detail was obtained where possible. Psychological formulations resulting from the LEDS were compared to formulations made by the clinical team that had assessed the patient; if a key stressor had been documented in the medical charts (even in little detail) this was counted as having been previously identified.

### Other assessments

IQ was estimated using the National Adult Reading Test (NART; Nelson & Willison, 1991). Anxiety and depression symptoms were assessed using the self-rated Hospital Anxiety and Depression Scale (HADS; Zigmond & Snaith, 1983) (CD and healthy controls only).

### Power calculation

A target sample size of 40 CD patients and 40 controls was chosen to enable detection of a difference in number of life events in a given time period with a standardized effect size of 0.57 with conventional levels of significance (0.05) and power (80%), which would be within the lower range of effect sizes in previously reported studies using the LEDS (House & Andrews, 1988; House *et al.* 1990; Craig *et al.* 1994; Harris *et al.* 1996; Hatcher & House, 2003).

### Analysis

The median/mean number of events per unit time (adjusted to annual rates to facilitate comparisons) were compared between groups using Mann–Whitney *U* tests as distributions were non-normal. Proportions of participants experiencing events were compared with χ^2^ tests, and ORs with 95% CIs were calculated.

## Results

### Subject characteristics

Forty-seven CD patients were recruited but four were excluded giving a total of 43 for analysis; one was subsequently diagnosed with myasthenia gravis, two had memory impairment and one patient had speech impairment leading to inability to complete the LEDS. Other CD patients reported poor memory but with sufficient time, provision of ‘time anchors’ (memorable times of year such as Christmas, birthdays or holidays) and prompts (from collateral histories) were able to give quite accurate dates of events. The three comparison groups did not significantly differ with respect to sex, age (matching criteria for controls), or marital status or social class. Depression scores were significantly (*p* = 0.017) higher in CD patients compared to healthy controls. Anxiety scores in CD were also higher, but not significantly so, compared to healthy controls. Mean scores for both groups for depression and anxiety were below thresholds for definite caseness (HADS scores <11). CD patients had significantly (*p* = 0.007) lower estimated IQs but did not differ for age of leaving education. (See Table 1 for full details.)

**Table 1.** Summary of subjects’ characteristics

Variable	CD	HC	DEP	CD *v.* HC *v.* DEP		CD *v.* HC		CD *v.* DEP	
	(*N* = 43)	(*N* = 28)	(*N* = 28)	Stats	*p*	Stats	*p*	Stats	*p*
Female (%)	34 (79.1)	20 (71.4)	22 (78.6)	χ^2^ = 0.63	0.73	χ^2^ = 0.54	0.46	χ^2^ = 0.003	0.96
Age, years (s.d.)	38.0 (10.5)	38.3 (8.4)	39.2 (10.75)	ANOVA	0.84	*t* = 0.11	0.91	*t* = 0.48	0.64
Estimated IQ (s.d.)	100.3 (12.8)	109.5 (11.9)	–	–	–	*t* = 2.80	0.007**	–	–
HADS Anxiety (s.d.)	10.6 (5.2)	8.1 (3.6)	–	–	–	*t* = −1.94	0.06	–	–
HADS Depression (s.d.)	8.3 (5.2)	5.1 (3.6)	–	–	–	*t* = −2.45	0.02*	–	–
Marital status (%)									
Single	6 (13.9)	8 (28.6)	10 (35.7)	χ^2^ = 9.54	0.05*	χ^2^ = 3.74	0.16		
Married	31 (72.1)	19 (67.9)	12 (42.9)					χ^2^ = 6.52	0.04*
Separated	6 (13.9)	1 (3.6)	6 (21.4)						
Social class^a^ (%)									
I/II	18 (41.9)	8 (28.6)	8 (28.6)	χ^2^ = 4.64	0.59	χ^2^ = 2.73	0.44	χ^2^ = 1.74	
III	10 (23.2)	6 (21.4)	8 (28.6)						0.63
IV	8 (18.6)	6 (21.4)	8 (28.6)						
V	6 (13.9)	8 (28.6)	4 (14.3)						

CD, Conversion disorder patients; HC, healthy controls; DEP, depression controls; *t*, Student's *t* test; HADS, Hospital Anxiety and Depression Scale

a Social class defined using standard UK (Registrar General) classification.

*p ⩽ 0.05, **p ⩽ 0.01.

### Events

Events were analysed in terms of both the presence of a single event and the mean number of events per epoch, as it is not known whether single stressors are likely to be relevant to CD or whether the potential influence of stressors is cumulative with a specific threshold.

The nature and timing of events were analysed as follows. Severity and escape were combined into four key groups to investigate event nature: (1) ‘All events’ (events of any severity), (2) ‘Severe events’, (3) ‘High escape events’, and (4) ‘Severe high escape events’. Three main time periods (epochs) were selected to assess temporal associations with CD onset: whole study, last 3 months and last month. For CD patients and healthy controls additional data were available for the last week and last 24 h allowing finer-grained analysis of timing. (See Tables 2 and 3 for full results.)

**Table 2.** Number of subjects with at least one life event per epoch in patients with conversion disorder (CD), healthy controls (HC) and depression (DEP)

Epoch/event type	Subjects with ⩾1 stressor per epoch (%)													
	CD (*N* = 43)		HC (*N* = 28)		DEP (*N* = 28)		CD *v.* HC				CD *v.* DEP			
	*n*	(%)	*n*	(%)	*n*	(%)	*p*	χ^2^	OR	95% CI	*p*	χ^2^	OR	95% CI
Whole study														
All	43	(100)	25	(89)	26	(93)	0.057	FET	1.12	0.99–1.27	0.152	FET	1.08	0.97–1.19
Severe	36	(84)	18	(64)	21	(75)	0.061	3.52	2.86	0.93–8.75	0.367	0.82	1.72	0.53–5.56
Escape	25	(58)	2	(7)	10	(36)	<0.001***	18.7	18.06	3.79–86.0	0.065	3.41	2.50	0.94–6.67
Severe escape	21	(49)	2	(7)	7	(25)	<0.001***	13.5	12.41	2.61–58.9	0.045*	4.03	2.87	1.01–8.13
Last 3 months														
All	34	(79)	21	(75)	18	(64)	0.688	0.16	1.26	0.41–3.89	0.169	1.89	2.10	0.72–6.10
Severe	30	(70)	5	(18)	11	(39)	<0.001***	18.3	10.61	3.31–34.1	0.011*	6.46	3.57	1.31–9.71
Escape	23	(54)	1	(3)	8	(29)	<0.001***	18.9	31.05	3.86–249.5	0.039*	4.28	2.87	1.04–7.94
Severe escape	21	(49)	0	(0)	6	(21)	<0.001***	19.4	–	–	0.020*	5.41	3.50	1.18–10.32
Last month														
All	32	(75)	13	(46)	13	(46)	0.017*	5.72	3.36	1.22–9.22	0.017*	5.72	3.36	1.22–9.26
Severe	24	(56)	5	(18)	6	(21)	0.001***	10.1	5.81	1.86–18.2	0.004**	8.22	4.63	1.56–13.70
Escape	23	(53)	0	(0)	4	(14)	<0.001***	22.2	–	–	0.001***	11.1	6.90	2.05–23.26
Severe escape	20	(47)	0	(0)	3	(11)	<0.001***	18.1	–	–	0.002**	9.92	7.25	1.90–27.78

OR, Odds ratio; CI, confidence interval; FET, Fisher's exact test.

Severe, score of 1 or 2 on long-term threat; Escape, score of 2 or 3 on escape rating.

Three-way comparison between groups (CD *v*. HC *v*. DEP) significant for all comparisons (*p* ⩽ 0.05) using χ^2^/FET).

*p ⩽ 0.05, **p ⩽ 0.01, ***p ⩽ 0.001.

**Table 3.** Rates of life events in conversion disorder (CD), healthy controls (HC) and depression (DEP)

Epoch/event type	Rates of event per year^a^												
	CD (*N* = 43)			HC (*N* = 28)			DEP (*N* = 28)			CD *v*. HC		CD *v*. DEP	
	Mdn	IQR	Mean	Mdn	IQR	Mean	Mdn	IQR	Mean	*U*	*p*	*U*	*p*
Whole study													
All	6	4	6.31	4	4	4.61	2	3	3.64	456.0	0.086	322.5	0.001***
Severe	1.5	2	2.13	0.5	1	1.04	1	2.5	1.71	396.0	0.014*	542.5	0.475
Escape	1	2	1.28	0	0	0.21	0	1	0.64	295.5	<0.001***	445.0	0.050*
Severe escape	0.5	1	0.95	0	0	0.21	0	0.8	0.39	360.5	<0.001***	425.0	0.021*
Last 3 months													
All	8	12	9.58	4	7	6.43	4	8	5.29	446.0	0.061	401.5	0.016*
Severe	4	8	4.47	0	0	0.71	0	4	2.29	262.0	<0.001***	423.0	0.024*
Escape	4	4	3.16	0	0	0.14	0	4	1.43	298.0	<0.001***	446.0	0.039*
Severe escape	0	4	2.70	0	0	0	0	0	1.00	308.0	<0.001***	429.0	0.018*
Last month													
All	12	24	13.4	6	12	8.57	0	12	8.14	417.0	0.020*	410.5	0.017*
Severe	12	12	6.70	0	0	2.14	0	0	3.00	373.5	0.002**	407.0	0.008**
Escape	12	12	6.70	0	0	0	0	0	2.14	280.0	<0.001***	375.5	0.002**
Severe escape	0	12	5.58	0	0	0	0	0	1.23	322.0	<0.001***	386.5	0.002**

Mdn, Median; IQR, interquartile range; *U*, Mann–Whitney *U* test.

Severe, scores 1 or 2 on long term threat; Escape, score of 2 or 3 on escape rating.

Three-way comparison between groups (CD *v*. HC *v*. DEP) significant for all comparisons (*p* ⩽ 0.05) using Kruskal–Wallis.

a Number of events in each epoch are converted to a rate over 1 year (‘annualized’) allowing direct comparisons between epochs of different lengths (note that epochs are not exclusive).

*p ⩽ 0.05, **p ⩽ 0.01, ***p ⩽ 0.001.

### Nature of events

CD patients had higher rates of ‘all events’ compared to both control groups for all epochs but the differences were not pronounced and were of variable, mostly low, significance. However, CD patients had significantly elevated rates of both severe life events and high escape events compared to both control groups and this was similar between measures of at least one event and mean numbers of events per epoch. Fifty-six per cent of CD patients had at least one severe event in the month before symptom onset – significantly more than 21% in depression cases (OR 4.63, 95% CI 1.56–13.70) and healthy controls (OR 5.81, 95% CI 1.86–18.2). In the same time period 53% of CD patients had at least one ‘high escape’ event – again significantly higher than 14% in depression cases (OR 6.90, 95% CI 2.05–23.6) and 0% in healthy controls.

Similar results are seen for total numbers of severe events per year in the last month; median = 12.0 (mean = 6.7) for CD – significantly more than depression cases (median = 0, mean = 3.0, *p* = 0.008) and healthy controls (median = 0, mean = 2.1, *p* = 0.002). Rates of high escape events per year are similarly elevated in CD patients (median = 12.0, mean = 6.7), significantly more than depression patients (median = 0, mean = 2.1, *p* = 0.002) and healthy controls (median = 0, mean = 0, *p* < 0.001). When severe events, which were also ‘high escape’, are considered, even greater group differences were observed for the three main epochs (all *p* ⩽ 0.001). Rates of under-reported events (of any type or severity) were low for the whole study period (mean = 0.26 in CD and 0.25 in both depression and healthy controls) with no significant group differences in any epoch.

### Timing of events

**Fig. 1.**
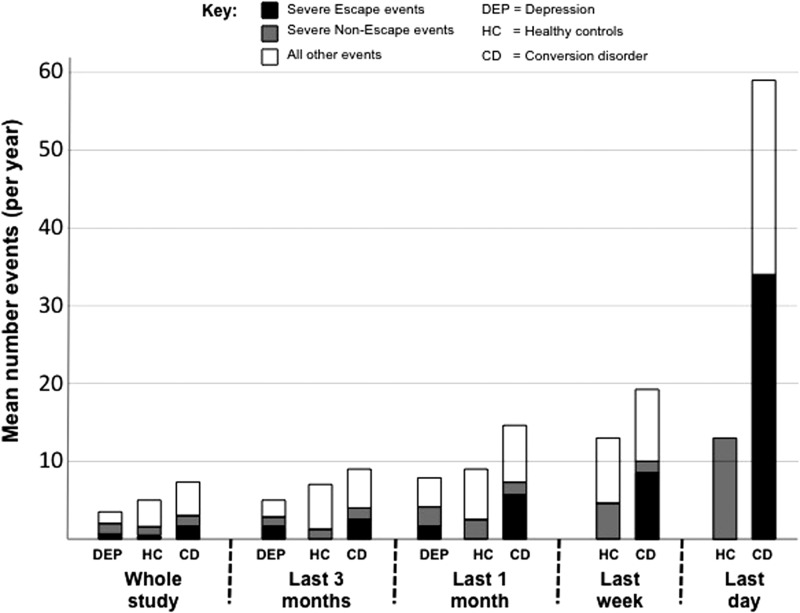
Total number of events during different epochs of the study. Note that epochs are not exclusive and the number of events in each epoch are converted to rate over 1 year (‘annualized’) allowing direct comparison between epochs of different lengths. Data on ‘last week’ and ‘last day’ epochs were not available for depression cases.

Increasing rates of events were seen for all groups with increasing closeness to symptom onset (or end of study period for healthy controls) as would be expected with recent effects. However, this increase is markedly more for CD than controls; Fig. 1 illustrates this visually by plotting mean numbers of events (adjusted to number/year allowing direct comparison between epochs). This is even more marked for the last week and last 24 h; in CD rates of all events/year rise to 19.4 in the last month and 59.1 in the last 24 h with an escalating proportion being severe events and/or high escape events.

### Difficulties

There were no significant increases in the number of severe difficulties between the groups. Difficulties of high escape were significantly (*p* ⩽ 0.004) more frequent in CD patients compared to healthy controls, but not depression cases, across all time periods.

### Sexual and physical abuse

During the interview, 22/43 (51.2%) CD patients reported previous abuse with 18 (41.9%) reporting sexual abuse and 10 (23.8%) reporting physical abuse. Rates of sexual, but not physical, abuse were significantly higher in CD patients compared to healthy controls. Female depression patients reported intermediate rates of sexual abuse (30.4%, 7/22), with similar rates of physical abuse (22.7%) as both CD patients and healthy controls. Comparative data are not available for male depression cases. (See Table 4 for details.)

**Table 4.** Rates of abuse in conversion disorder (CD), healthy controls (HC) and depression (DEP)

Variable	CD (*N* = 43)		HC (*N* = 28)		DEP (*N* = 28)		CD *v.* HC		CD *v*. DEP	
	*n*	(%)	*n*	(%)	*n*	(%)	Stats	*p*	Stats	*p*
Sexual abuse										
Female	18	(52.9)	3	(15.0)	7	(31.8)	χ^2^	0.006**	χ^2^	0.12
Male	0	(0)	1	(12.5)	n.a.		FET	0.47	n.a.	
Total	18	(41.9)	4	(14.2)	n.a.		χ^2^	0.014*	n.a.	
Physical abuse										
Female	9	(26.5)	3	(15.0)	5	(22.7)	FET	0.50	χ^2^	0.75
Male	1	(11.1)	3	(37.5)	n.a.		FET	0.29	n.a.	
Total	10	(23.2)	6	(21.4)	n.a.		χ^2^	0.86	n.a.	

FET, Fisher's exact test; n.a., data not available.

*p ⩽ 0.05, **p ⩽ 0.01.

### Aetiological formulation/key events

Key events and/or a psychological formulation were identifiable for almost all CD patients (39/43, 91%). In terms of types of stressor certain gender-specific themes emerged. For the 33 females, events involved seeing or hearing about a previous abuser (*n* = 12), marriage/partner events (*n* = 5) and termination of pregnancy (*n* = 2). For the 10 males, work events (six patients) and accidents with others potentially to blame (four patients) predominated. For the majority (38/43, 88.4%) of patients the key event(s) had not been documented in the clinical notes and/or correspondence during clinical care before entering the study.

## Discussion

We found significantly elevated rates of both severe life events and high escape events in CD *v.* controls that became more marked with increasing closeness to symptom onset. Higher rates of sexual abuse were also reported in CD *v.* controls and in one third of females abuse was relevant to life events around disorder. We found no evidence for ‘repression’ (of the severity of life events). The majority of potentially aetiologically relevant life events had not been picked up by routine clinical assessments. However, in a significant minority (10%) of CD cases no severe life events were identifiable.

### Limitations

This study is limited by several potential biases. First, CD patients were recruited from specialist services and are therefore likely to be relatively severe and/or have shown little initial recovery, thereby limiting generalizability. Second, interviewers were unblinded to case-control status, although the consensus panels rating events were. Furthermore, healthy controls, despite efforts to minimize this, had slightly shorter times (mean 1.96 years compared to 2.42 years) between study period start dates and interview dates leading to potential recall bias. A similar recall bias could occur due to memory deficits in CD which are relatively commonly reported and for which there is some evidence of actual deficit (Brown *et al.* 2013), although removing such recall biases would only increase the differences we found. Detecting abuse with the LEDS could result in underestimations (e.g. unwillingness to disclose such sensitive information during face-to-face interviews) or overestimations (e.g. false memories as confirmatory evidence not obtained) although these potential biases also occur with other detection methods (Jonas *et al.* 2010).

In terms of specificity of the results, although psychiatric controls were available they were from an earlier study so cannot be considered fully ‘matched’ like the healthy controls and there is not comparable data on IQ and HADS scores. However these depression cases were interviewed using the Present State Examination (Wing *et al.* 1974), with supplementary questions to enable research diagnostic criteria (Spitzer *et al*. 1978) and DSM characterizations of neurotic, psychotic and endogenous subtypes, and were from the same geographical area and similar in age, sex and key socio-economic variables to the CD patients. The healthy controls might not have been perfectly matched for all variables, as evidenced by the significantly higher mean IQ in controls (109.5) compared to CD patients (100.3) which is likely to result from a bias among the healthy individuals accepting invitations to take part in the study. Finally, there was no neurological control group and elevated rates of life events have been found in stroke (House *et al.* 1990) and multiple sclerosis (Grant *et al.* 1989), albeit at considerably lower rates than found in CD in this study with similar methods (LEDS). It should also be noted that stress is associated with the onset or exacerbation of many other neurological disorders, for example migraine (Peroutka, 2014) and epilepsy (Wassenaar *et al*. 2014), as well many other non-neurological disorders, for example myocardial infarction (Wei *et al*. 2014) and psoriasis (Hunter *et al*. 2013).

### Implications

#### The psychological model

Although our finding of elevated rates of stressors in the year before onset of CD supports the psychological model it of course does not establish aetiological relevance, especially as our study highlights that stressors are relatively common in controls. In any given individual chance could therefore account for the presence of a stressor, and evidently this would become more likely with increasing time between stressor and symptom onset. However, the rapidly escalating rates of severe events we found in CD, but not controls, with increasing proximity to symptom onset provides support for aetiological relevance.

Life events, both before and around the time of onset, form the basis psychological formulations. The potentially chance nature of stressors around symptom onset is an important reminder that hastily considered formulations will make little sense to patients and only serve to move them away from, not towards, a psychological model. However, the method of identifying psychological formulations in this study is validated by a subgroup of patients from this study showing differential brain activations when recalling these events, compared to other equally severe events, which is consistent with contemporary neurobiological hypotheses of the disorder (Aybek *et al.* 2014). It must be emphasized that the importance of life events can only be gauged through detailed appreciation of context – as it is in the LEDS. A seemingly innocuous event, e.g. a phone call, might provide news of the reappearance of, or somehow prompt the memory of, a past abuser and reactivate previous traumas or prompt a major change in work status. It should also be emphasized that many, and possibly even all, of the stressors identified in the year before, or even immediately before onset might not be causally related to the disorder and could have occurred at any time in the patient's life (or indeed in any control's life). However, despite this important caveat, it was possible to make a very convincing psychological formulation for the majority of patients on the basis of the stressors identified in the year, and particularly the few weeks and months, before the onset of the symptoms.

It is also important to note that despite the thoroughness of the LEDS severe stressors were not identifiable in a significant minority of patients (4/43, 9%), consistent with previous studies (Duncan & Oto, 2008). This supports the recent downgrading of the identification of such stressors from an essential diagnostic criterion (APA, 2013). However, LEDS assessments identified over four times more stressors relevant to psychological formulations than standard clinical interviews and this makes a compelling case that more, not less effort, should be put into their identification as this might support the diagnosis or be of therapeutic use.

#### Freudian theories

The particular association of CD with escape events supports Freud's theory of secondary gain. The aforementioned limitation our sample being likely to have relatively chronic patients might be particularly relevant to the secondary gain findings in that this might be a factor impeding recovery rather than being of relevance to the initial symptoms. It should also be noted that a significant proportion of CD patients had no escape events so even if it is an aetiological factor it is likely just one of many possible factors, albeit a potentially potent one.

Using rates of ‘under-reporting’, we saw no evidence of clinical repression in this sample, albeit with coarse methodology. If there were events that were repressed to the extent that they were unavailable to conscious recollection, different methodologies would be needed (e.g. collateral histories) to quantify these. We have recently explored whether patients with CD are able to use memory suppression in an experimental context, as defined by contemporary cognitive science, to a greater extent than controls and have been unable to confirm this (Brown *et al.* 2013).

We found support for the importance of sexual abuse as a remote risk factor for CD through elevated rates of abuse and from the subgroup of patients for whom this abuse was clearly re-activated around symptoms onset. It should be noted that such abuse is relatively common in the general population and, as a particularly potent stressor, a relatively non-specific risk factor for psychiatric disorder (Jonas *et al.* 2010). However, our findings are consistent with previous studies showing an interaction between sexual abuse, family dysfunction and the seizure variant of CD (Salmon *et al.* 2003). There is also evidence that the nature and chronicity of abuse might be important. For example, findings from a large controlled study of abuse in motor CD which reported particularly strong associations with both incestual and chronic sexual abuse (Roelofs *et al.* 2002).

#### Potential mechanism(s)

*‘*Stress-diathesis’ models, which propose that individuals have variable susceptibility (or thresholds) for the development of disorders (Rosenthal, 1963), could resolve the apparent paradox of the key findings in this study that stressors seem relevant to some, but not all, CD patients. Factors influencing an individual's threshold may vary from purely ‘biological’ factors such as the physiological stress response (e.g. hypothalamic-pituitary axis function) through to psychological factors (e.g. personality, coping strategy and illness models) all of which are likely shaped by a combination of genetic programming and environmental factors such as childhood experiences. There is preliminary evidence that in the seizure variant of CD basal cortisol levels are elevated and that this correlates with levels of childhood abuse (Bakvis *et al.* 2010).

There has been more work in the related condition of post-traumatic stress disorder (PTSD) where ‘gene x environment’ (GxE) studies have indicated that HPA axis variation, and the genes controlling it, interact with childhood abuse to increase risk of developing the disorder (Binder *et al.* 2008). Therefore ‘high-risk’ individuals, via genetic variants and/or exposure to childhood trauma, could develop PTSD after a relatively minor recent trauma whereas ‘low-risk’ individuals would require a major trauma to trigger the disorder. Similar processes may apply in CD. However, unless it can be shown that specific types of stressors cause different psychiatric disorders (which seems unlikely), it is possible that severe events of escape potential are particularly potent in shaping expression of psychiatric distress in a person vulnerable to CD.

## Conclusion

Using rigorous psychosocial methodology we found evidence supporting the psychological model of CD and for some aspects of the Freudian model. The study suggests that aetiologically, and possibly also therapeutically, relevant stressors are often missed in routine clinical practice and this is an important reminder of the continued importance of thorough psychiatric evaluation.
